# The Impact of an 8-Week Deliberate Practice Intervention on Coincidence Anticipation Timing and Long-Term Retention in Youth Female Volleyball Players

**DOI:** 10.3390/children13060822

**Published:** 2026-06-17

**Authors:** Evangelia Amprasi, Nerantzoula Koufou, Ioannis Trigonis, Ioannis Tsartsapakis, Aglaia Zafeiroudi, Olga Kouli

**Affiliations:** 1 Department of Physical Education & Sport Science, Democritus University of Thrace, 69100 Komotini, Greece; eamprasi@phyed.duth.gr (E.A.); nkoufou@phyed.duth.gr (N.K.); itrigon@phyed.duth.gr (I.T.); okouli@phyed.duth.gr (O.K.); 2 Department of Physical Education & Sport Science, Aristotle University of Thessaloniki, 62122 Serres, Greece; 3 Department of Physical Education & Sport Science, University of Thessaly, 42100 Trikala, Greece; azafeiroudi@uth.gr

**Keywords:** coincidence anticipation timing, deliberate practice, motor learning, volleyball, neuroplasticity, predictive control, retention, youth sports, interceptive skills

## Abstract

**Highlights:**

**What are the main findings?**

- An 8-week deliberate practice program significantly improved Coincidence Anticipation Timing (CAT) accuracy in girls aged 8–10, with gains being notably more pronounced at higher stimulus velocities (10 mph).
- The experimental group demonstrated robust long-term retention of timing skills, maintaining superior accuracy during a two-month post-intervention test compared to the control group.

**What are the implications of the main findings?**

- The results suggest that targeted, high-feedback training can accelerate the refinement of predictive motor control models during the critical developmental stage of late childhood.
- Coaches should integrate structured timing interventions as foundational components for interceptive sports, capitalizing on the heightened capacity for sensorimotor adaptation during the pre-adolescent period.

**Abstract:**

Background/Objectives: In interceptive sports like volleyball, the ability to accurately time an action relative to a moving object (Coincidence Anticipation Timing—CAT) is critical. This study investigated the effects of a structured 8-week deliberate practice intervention on CAT accuracy and its long-term retention in young female athletes. Methods: Thirty-two female volleyball players (aged 8–10 years) were randomly assigned to an Experimental Group (EG, *n* = 16) and a Control Group (CG, *n* = 16). The EG underwent a specialized 8-week training program focusing on progressive cognitive load and immediate knowledge of results, while the CG followed standard volleyball training. A single-blind assessor measured CAT at two velocities (5 mph and 10 mph) using the Bassin Anticipation Timer at three time points: pre-test, post-test, and a 2-month retention test. Results: ANCOVA revealed a significant group-by-time interaction (*p* < 0.001), with the EG demonstrating a substantial reduction in absolute timing error. The effect size was markedly higher at 10 mph (partial η^2^ = 0.400) compared to 5 mph (partial η^2^ = 0.197). Crucially, the EG maintained their performance gains during the retention test (*p* < 0.05), whereas the CG showed no significant improvement over time. Conclusions: Targeted deliberate practice effectively enhances temporal prediction accuracy in children, likely by facilitating a shift from reactive to predictive motor control. The robust retention of these skills underscores the significant neuroplasticity of the 8–10 age window, suggesting that early foundational timing interventions provide long-lasting benefits for athletic development.

## 1. Introduction

Volleyball is a high-intensity, open-skill team sport that imposes extraordinary visual-perceptual and decision-making demands [1,2]. Unlike closed-skill environments, volleyball requires athletes to operate within a context of rapid, unpredictable ball trajectories and constantly shifting opponent configurations [3,4]. Success in all game phases is contingent upon the athlete’s ability to process dynamic visual information under extreme time pressure [5,6]. Central to this process is robust perceptual-motor coupling, where movements are continuously reorganized in response to perceived environmental information [7,8]. In such a fast-paced interceptive sport, the capacity for anticipation, predicting future events based on partial information, becomes the primary determinant of elite performance [1,9].

A critical component of this anticipatory capacity is Coincidence Anticipation Timing (CAT), defined as the ability to predict the arrival of a moving stimulus at a specific target point to initiate a synchronized motor response [1]. CAT serves as the foundational mechanism for interceptive actions such as hitting, catching, and blocking, where millisecond-level spatiotemporal accuracy is required [10,11]. Research indicates that CAT performance is a significant discriminator of expertise, with expert athletes demonstrating superior timing accuracy and lower variability compared to novices [12,13,14]. In volleyball specifically, the integration of visuomotor speed and reaction capacity through CAT enables players to effectively counter high-velocity attacks and complex sets [11,15].

The developmental trajectory of CAT and its associated perceptual-motor skills undergoes a profound transition during late childhood, specifically between the ages of 8 and 10 [16,17]. This period is considered a “critical window” for the refinement of the vision-for-action pathway (the dorsal stream) [17]. While basic visual sensory functions are relatively mature by age 6, the motor system’s contribution to multisensory integration continues to evolve significantly until approximately age 10 [16]. During this stage, children begin to demonstrate more adult-like weighting of visual and proprioceptive cues, although their temporal prediction and timing stability remain significantly more variable [18,19]. Optimizing CAT during this developmental phase is essential, as neural networks in the precentral gyrus and supplementary motor cortex become increasingly specialized for visuomotor control [19,20].

Theoretical frameworks such as ecological dynamics and perception-action coupling provide a robust basis for understanding how these skills are acquired. According to the ecological perspective, skill emergence involves becoming “perceptually attuned” to the specifying information available in the environment [7,21]. Experts learn to exploit kinematic cues, such as an opponent’s postural orientation, to guide their motor responses, allowing for a reciprocal interaction where perceptual information guides movement, and movement reorganizes perceptual pickup [1,2,8,13,22].

For young athletes, the challenge lies in learning to decouple irrelevant information while focusing on high-validity cues that support successful interception [23,24].

A fundamental mechanism underlying CAT is the estimation of Time-to-Contact (TTC), often explained through the optical variable tau (τ) [25]. However, 8–10-year-old children have not yet attained adult-like sensitivity to this optical expansion (looming). Studies utilizing radial optic flow indicate that children in this age range exhibit lower sensitivity and slower latency in detecting approach rates [25,26]. Furthermore, the maturation of motion-onset visual evoked potentials (VEPs) and the magnocellular pathway continues into late childhood, resulting in less precise TTC estimates [27,28,29]. This developmental immaturity highlights the necessity for structured interventions to enhance TTC estimation efficiency.

Regarding the acquisition of these skills, training interventions utilizing augmented feedback are significantly more effective than simple repetition [30,31]. The provision of “knowledge of results” (KR) and “knowledge of performance” (KP) accelerates learning and enhances long-term retention [32,33]. In tasks requiring high-fidelity timing like CAT, seeing one’s error quantified in milliseconds serves as a powerful error-correction signal that facilitates rapid visuomotor adaptation [30,34]. Youth athletes are highly responsive to precise, real-time feedback, which improves accuracy and boosts intrinsic motivation [31,35,36].

The theory of “deliberate practice,” proposed by Ericsson et al. [37], provides a rigorous framework for such interventions. Deliberate practice is defined as a highly structured, effortful activity designed to improve performance through focused repetition and immediate feedback [37,38,39]. Unlike “deliberate play,” deliberate practice requires the learner to actively monitor performance and correct specific weaknesses [40]. While widely studied in general motor skill acquisition, its specific impact on the “micro-structure” of perceptual skills like CAT in young, developing athletes remains under-explored [39].

Despite the known plasticity of the developing brain, there is a paucity of research investigating the effects of deliberate practice on CAT specifically within the 8–10-year-old age group. Existing literature often lacks longitudinal evidence and focuses on general motor skills rather than specialized interceptive timing. Furthermore, data regarding the long-term retention of CAT improvements in children are lacking. Therefore, this study aims to address this gap by investigating the impact of an 8-week deliberate practice intervention on CAT performance and retention in young female volleyball players. We hypothesized that the experimental group would demonstrate significantly greater reductions in absolute timing error across multiple stimulus speeds (5 mph and 10 mph) compared to the control group. Furthermore, we hypothesized that these performance gains would be robustly maintained over a two-month retention period, leveraging the unique developmental sensitivity of this age window.

## 2. Materials and Methods

### 2.1. Study Design

This study employed a randomized controlled experimental design with three assessment time points: pre-test, post-test, and retention test. Specifically, the study utilized a single-blind approach, where the primary evaluator responsible for data collection was kept entirely unaware of the participants’ group assignments throughout the pre-test, post-test, and retention phases. The purpose of the design was to examine the short- and long-term effects of a deliberate practice intervention on Coincidence Anticipation Timing (CAT) in children aged 8–10 years. After baseline testing, participants were randomly assigned to either an Experimental Group (EG), which received an 8-week CAT-focused deliberate practice program, or a Control Group (CG), which continued regular volleyball training without CAT-specific activities. CAT performance was evaluated before the intervention, immediately after its completion, and again two months later to assess retention in the absence of continued practice.

### 2.2. Participants

Thirty-two beginner volleyball players (*n* = 32), all girls aged 8–10 years (mean +/− SD: 9.22 +/− 0.80 years), were recruited from a volleyball club in Komotini, Greece. Recruitment followed a self-selection process: parents who expressed interest in the study were contacted and informed about the study aims, procedures, and time commitments. Children were screened for eligibility prior to enrollment.

Inclusion criteria were: (a) female sex, (b) age between 8 and 10 years at study onset, (c) current participation in organized volleyball training, and (d) ability to attend all intervention and testing sessions. Exclusion criteria included any clinically significant neurological, musculoskeletal, visual, or other health condition that could interfere with safe participation in the intervention or testing procedures.

After baseline assessment, participants were randomly assigned to either the Experimental Group (EG; *n* = 16) or the Control Group (CG; *n* = 16) using simple randomization performed by an investigator not involved in the training or testing. Parents or legal guardians provided written informed consent, and children provided verbal assent. The study was approved by the Ethics Committee of Democritus University of Thrace (protocol 6 2020 M.A.) and conducted in accordance with the Declaration of Helsinki.

An a priori power analysis was conducted using G*Power 3.1 (*F*-tests, ANCOVA: fixed effects, main effects and interactions) to determine the appropriate sample size prior to the intervention. Based on the focused and intensive nature of the deliberate practice program, a large effect size was anticipated. Assuming an expected effect size of *f* = 0.52, an alpha level of 0.05, a desired statistical power of 0.80, two groups, and one covariate, the required total sample size was calculated to be 32 participants. Therefore, our recruited sample of *N* = 32 was strictly adequate to detect the anticipated main and interaction effects. Exactly 32 participants were initially recruited, and a per-protocol analysis was conducted on all 32 who completed all phases. This extraordinary 100% adherence rate was successfully managed because the intervention was formally integrated into the club’s mandatory training schedule during a competitive period, and the gamified nature of the CAT drills maintained exceptionally high motivation among the young athletes.

### 2.3. Procedures

A pre-test was conducted to assess coincidence anticipation timing (CAT) before the start of the intervention period. After obtaining written informed consent from parents and assent from the children, all eligible players completed the CAT assessment under standardized conditions. Subsequently, participants were randomly allocated, using a computer-generated randomization list, to either the experimental group (EG) or the control group (CG), with 16 players in each group.

Both groups continued to attend their regular volleyball training sessions at the club throughout the 8-week study period (two sessions per week, 60 min per session). Regular training consisted of general technical and tactical volleyball activities (e.g., passing, serving, basic offensive and defensive drills, small-sided games) and did not include any structured CAT-specific exercises or explicit timing drills using light-based devices. Coaches of the CG were explicitly instructed to avoid introducing new drills that focused on coincidence timing or reaction to moving visual stimuli during the study period.

For the EG, a 24-min CAT-focused deliberate practice block was embedded at the beginning of each regular training session, replacing an equivalent amount of generic technical work so that total training volume was comparable between groups. The CG completed the usual technical-tactical content during the same time window. At the end of the 8-week intervention, all participants completed a post-test CAT assessment under identical conditions to the pre-test. A retention test was then administered two months after the end of the intervention period. During this two-month interval, both groups followed only their standard volleyball training schedule without any additional specialized timing or deliberate practice interventions. This period allowed for the assessment of long-term skill permanence independent of immediate training effects.

To monitor protocol fidelity, coaches in both groups completed standardized training logs after each session, documenting the type and duration of activities performed. In addition, a member of the research team conducted unannounced observations of one training session every two weeks for each group to verify adherence to the planned content and to ensure that no CAT-specific drills were introduced in the CG. Sessions with adherence below 90% to the planned structure were discussed with the coach and corrective feedback was provided.

During the two-month interval between the post-test and the retention test, both groups followed their standard volleyball training schedule without any additional specialized timing or deliberate practice interventions. This period allowed for the assessment of long-term skill permanence independent of immediate training effects.

**Figure 1:**
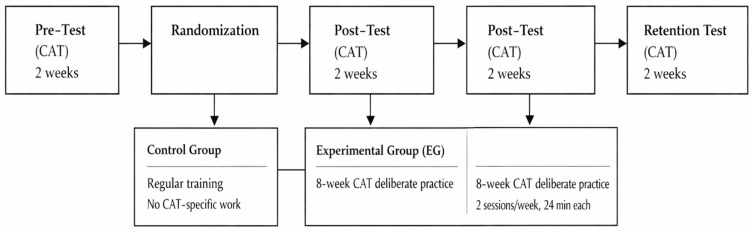
Study design. Schematic representation of the study flow and experimental design, detailing the timeline for pre-test assessment, random allocation into groups, the 8-week intervention phase, post-test assessment, and the 2-month retention test. CAT = Coincidence Anticipation Timing; EG = Experimental Group; CG = Control Group.

The overall study design is illustrated in Figure 1.

### 2.4. Intervention

The deliberate practice intervention for the EG was designed as a structured, feedback-rich program targeting improvement in CAT while maintaining age-appropriate physical and cognitive demands. In strict alignment with Ericsson’s framework, the intervention was operationally defined and executed through three core pillars of deliberate practice: (a) precise, individualized goal-setting adjusted dynamically to the children’s developmental stage; (b) expert coaching accompanied by continuous, high-density augmented feedback; and (c) sustained, high-concentration effort within brief, focused training intervals designed to prevent cognitive or physical fatigue. Each CAT-focused session lasted 24 min and was delivered twice per week over 8 consecutive weeks, integrated into the regular volleyball practice on the court.

Each session followed a consistent structure: (a) 4 min of dynamic warm-up with simple ball-handling activities (e.g., light jogging, ball tosses, catching and passing in place) to prepare the visual–motor system; (b) 16 min of CAT-specific drills; and (c) 4 min of low-intensity stretching and recovery. The 16 min CAT block consisted of four 4 min exercises that emphasized synchronizing a motor response with the arrival of a moving stimulus. Examples of ecologically valid drills included: (1) catching a ball thrown by a partner at a predefined target line (“catch the ball in the air”); (2) intercepting a ball rebounding from a wall at different distances; and (3) responding to a rolling ball crossing a marked zone. Additionally, to introduce stimulus variety, simple light-based reaction tasks (e.g., touching randomly illuminated portable pods) were occasionally incorporated as a supplementary activity (comprising <10% of total training time). Crucially, these supplementary tasks involved unpredictable, multi-directional spatial reactions, which fundamentally differ from the linear, constant-velocity tracking required by the Bassin Anticipation Timer. The order of the exercises was randomized across sessions to reduce predictability and maintain engagement. Task difficulty and progression were systematically manipulated across the 8 weeks. During weeks 1–2, drills were performed at slower ball speeds and with highly predictable trajectories to allow familiarization with the timing demands. In weeks 3–4, ball speed and distance were gradually increased, and variability in trajectory (e.g., slight changes in height or angle) was introduced. In weeks 5–6, more complex conditions were added, such as responding to balls delivered from different directions or including simple decision elements (e.g., catching only balls of a specific color). In weeks 7–8, the most challenging conditions were used, combining higher speeds, increased variability, and occasional dual-task elements (e.g., calling out a number or color while performing the interception) to increase cognitive load while preserving the core CAT requirement.

Feedback was provided systematically throughout the intervention. After each trial, players received immediate knowledge of results (KR) regarding the success or error of their timing (e.g., “too early”, “too late”, “on time”), and, when using timing devices, approximate timing error in milliseconds was communicated. In addition, knowledge of performance (KP) was offered every few trials, focusing on specific aspects of movement execution (e.g., starting movement earlier, maintaining visual fixation on the ball, coordinating arm and leg actions). At the end of each session, the coach briefly summarized individual progress and highlighted specific goals for the next session, reinforcing the deliberate, goal-oriented nature of practice.

To ensure fidelity of the intervention, all CAT sessions were delivered by the same coach, who received prior training on the protocol and a written manual describing each drill, its objectives, and progression criteria. After each session, the coach completed a checklist indicating which exercises were performed, the parameters used (e.g., distance, speed, decision elements), and any deviations from the planned structure. A second member of the research team observed approximately 20% of the sessions using a standardized observation form to verify adherence to the protocol and the consistent provision of feedback. Overall adherence above 90% was considered acceptable for fidelity purposes.

### 2.5. Outcome Measures

Coincidence Anticipation Timing (CAT) was assessed using the Bassin Anticipation Timer (Model 35575, Lafayette Instrument Company, Lafayette, IN, USA.), a widely used and psychometrically validated device for measuring temporal prediction accuracy in interceptive tasks. The apparatus consists of a 1.5-m linear runway containing 64 sequentially illuminated LEDs that simulate the motion of an approaching object. A handheld response switch, operated with the participant’s dominant hand, records the moment at which the child anticipates the arrival of the moving light at the terminal LED.

Testing was conducted individually in a quiet indoor space under standardized lighting and environmental conditions. Participants were seated at a fixed distance of 2 m from the center of the runway, with the terminal LED positioned at eye level to ensure consistent visual angles across trials. Before each trial, a yellow warning light was illuminated for 1.5 s, followed by the sequential activation of the red LEDs at one of two preset velocities (5 mph and 10 mph). Participants were instructed to press the response switch at the exact moment they perceived the moving light to reach the final LED.

Each participant completed two familiarization trials at each speed to minimize learning effects, followed by ten recorded trials per speed. The primary outcome variable was absolute timing error (in milliseconds), calculated as the absolute difference between the participant’s response time and the actual arrival time of the terminal LED. Absolute error is considered the most appropriate index of CAT performance in children, as it reflects accuracy independently of response direction (early vs. late). For each speed, the mean absolute error across the ten recorded trials was used in the statistical analyses. To ensure terminological consistency throughout this manuscript, ‘absolute timing error’ (or simply ‘absolute error’) serves as the precise quantitative metric used to operationalize and measure ‘timing accuracy,’ which in turn directly reflects the participants’ underlying capacity for ‘temporal prediction’ during the coincidence anticipation timing (CAT) task.

The Bassin Anticipation Timer has demonstrated high test–retest reliability (*r* = 0.94) and strong construct validity for assessing perceptual-motor timing in youth populations. All testing sessions were administered by the same trained evaluator, who was blinded to the participants’ group allocations (experimental vs. control) to ensure objective data collection and minimize potential assessor bias. Furthermore, this evaluator was not involved in the implementation of the 8-week deliberate practice intervention.

### 2.6. Statistical Analysis

All statistical analyses were performed using IBM SPSS Statistics, version 29.0 (IBM Corp., Armonk, NY, USA). Descriptive statistics (means and standard deviations) were calculated for all variables. The level of statistical significance was set at α = 0.05 for all tests.

Prior to inferential analyses, data were screened for outliers and missing values. The assumptions of normality, homogeneity of variances, linearity, and homogeneity of regression slopes were examined using Shapiro–Wilk tests, Levene’s tests, inspection of residual plots, and tests of interaction between the covariate and group, respectively. While the majority of statistical assumptions were met, violations of the homogeneity of variances (indicated by Levene’s tests in certain conditions) were addressed using robust statistical methods.

To compare CAT performance between groups while controlling for baseline differences, separate analyses of covariance (ANCOVA) were conducted for each stimulus speed (5 mph and 10 mph) and each post-intervention time point (post-test and retention test). In all ANCOVA models, the dependent variable was mean absolute CAT error at the corresponding time point, the fixed factor was group (EG vs. CG), and the covariate was the respective pre-test CAT score. This approach allowed adjustment for individual differences in baseline performance and provided a more precise estimate of the intervention effect.

In addition, within-group changes over time (pre-test to post-test and pre-test to retention) were explored using paired-samples *t*-tests for descriptive purposes, with Bonferroni adjustment applied to control for multiple comparisons. Effect sizes for between-group differences in the ANCOVA models were reported using partial eta squared (ηp^2^), interpreted according to Cohen’s guidelines (0.01 = small, 0.06 = medium, 0.14 = large). For *t*-tests, Cohen’s d was calculated.

To ensure the robustness of the ANCOVA models against the aforementioned violations of homoscedasticity (where Levene’s *p* < 0.05), robust bootstrap sensitivity analyses with 1000 resamples were executed. Robust 95% percentile confidence intervals (CIs) for the unstandardized parameter estimates (B) were computed and reported for all primary effects to guarantee parameter stability and quantify the precision of the findings.

## 3. Results

### 3.1. CAT Absolute Error Across Groups and Time Points

A total of 32 children successfully completed all training and assessment phases, yielding a 100% adherence and retention rate for the study. Descriptive statistics for CAT absolute error (measured in milliseconds) at both 5 mph and 10 mph across the pre-test, post-test, and two-month retention test are presented in Table 1. Initial visual inspection of the raw means indicates a trajectory of improvement in the Experimental Group (EG) following the intervention, while the Control Group (CG) maintained relatively stable error rates. Across both stimulus speeds and post-intervention time points, the EG consistently exhibited lower adjusted absolute timing error than the CG.

### 3.2. Post-Test CAT Performance at 5 mph

To evaluate the immediate impact of the deliberate practice intervention at the slower stimulus speed (5 mph), an ANCOVA was conducted, controlling for baseline CAT performance. As detailed in Table 2, the analysis revealed a statistically significant main effect of group on post-test absolute error, *F*_(1, 29)_ = 7.122, *p* = 0.012, partial η^2^ = 0.197 (indicating a large effect size). The pre-test covariate was a strong and significant predictor of post-test performance, *F*_(1, 29)_ = 39.091, *p* < 0.001, partial η^2^ = 0.574.

**Figure 2:**
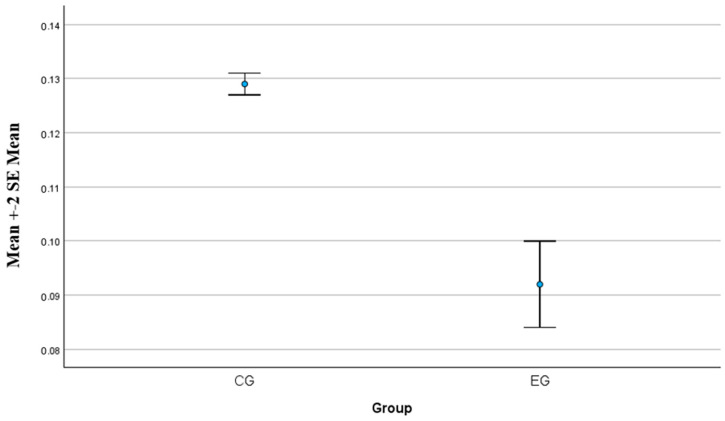
Adjusted means (±SE, Standard Error) for the Experimental Group (EG) and Control Group (CG) in the 5 mph (miles per hour) condition at post-test, controlling for baseline pre-test scores.

The adjusted marginal means confirmed superior temporal prediction accuracy for the EG (96 +/− 9 ms) compared with the CG (128 +/− 9 ms). Although Levene’s test indicated a modest violation of the homogeneity of variances assumption (*p* = 0.028), the robustness of the group effect was confirmed via bootstrap sensitivity analysis (1000 resamples, B = −32 ms, Bootstrap 2-tailed *p* = 0.032, Robust 95% CI: −57 to −9 ms). The adjusted means for this condition are illustrated in Figure 2.

### 3.3. Post-Test CAT Performance at 10 mph

Under the more demanding, higher-velocity condition (10 mph), the intervention yielded an even more pronounced effect. The ANCOVA demonstrated a highly significant main group effect on post-test absolute error, *F*_(1, 29)_ = 19.336, *p* < 0.001, partial η^2^ = 0.400 (a very large effect size). The pre-test covariate remained a powerful predictor of the outcome, *F*_(1, 29)_ = 76.906, *p* < 0.001, partial η^2^ = 0.726. Levene’s test indicated unequal variances (*p* = 0.002).

**Figure 3:**
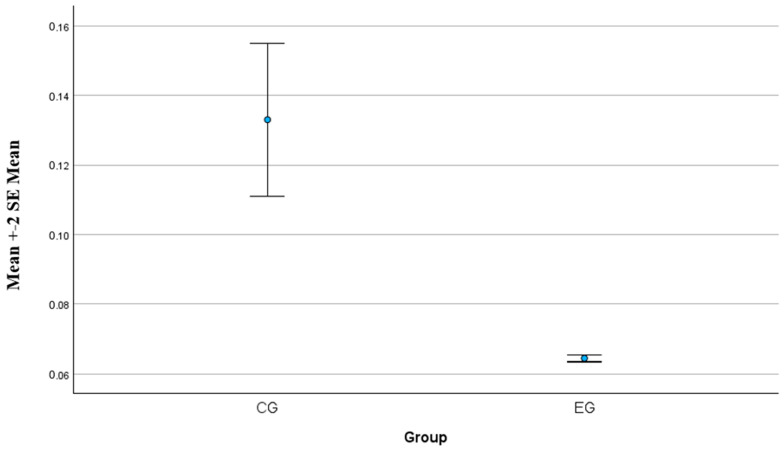
Estimated marginal means (±SE, Standard Error) for the Experimental Group (EG) and Control Group (CG) in the 10 mph (miles per hour) condition at post-test, controlling for baseline pre-test scores.

The adjusted means heavily favored the EG (64 +/− 9 ms) over the CG (122 +/− 9 ms), demonstrating that the deliberate practice protocol substantially enhanced the ability to process faster stimulus trajectories. Although Levene’s test indicated a violation of homoscedasticity (*p* = 0.002), the robustness of the group effect was confirmed via bootstrap sensitivity analysis (1000 resamples), yielding a significant robust parameter estimate (*B* = −58 ms, Bootstrap 2-tailed *p* = 0.006, Robust 95% CI: −87 to −34 ms). Corresponding adjusted means are shown in Figure 3.

### 3.4. Retention Test at 5 mph

To assess whether the perceptual-motor adaptations were sustained after a two-month period without CAT-specific training, retention data were analyzed. At 5 mph, the significant group effect persisted, *F*_(1, 29)_ = 13.179, *p* = 0.001, partial η^2^ = 0.312. The covariate continued to be a strong predictor, *F*_(1, 29)_ = 37.883, *p* < 0.001, partial η^2^ = 0.566, and Levene’s test was non-significant (*p* = 0.109), confirming the equality of error variances.

The adjusted marginal means highlighted the sustained superiority of the EG (88 ± 8 ms) compared with the CG (130 ± 8 ms), indicating successful long-term retention of the acquired timing skills.

### 3.5. Retention Test at 10 mph

Similarly, the retention test at the 10 mph condition revealed a robust and significant group effect, *F*_(1, 29)_ = 14.189, *p* < 0.001, partial η^2^ = 0.329. The pre-test score remained highly predictive of retention performance, *F*_(1, 29)_ = 65.127, *p* < 0.001, partial η^2^ = 0.692. Levene’s test indicated unequal variances (*p* = 0.024).

The adjusted means demonstrated that the EG maintained its performance advantage (65 +/− 9 ms) over the CG (114 +/− 9 ms) well beyond the cessation of the targeted training. Despite the mild violation of variance homogeneity, the bootstrap sensitivity analysis confirmed the stability of the intervention effect (*B* = −49 ms, Bootstrap 2-tailed *p* = 0.038, Robust 95% CI: −80 to −24 ms).

### 3.6. Summary of Findings

In summary, across all evaluated speeds and temporal milestones, the EG consistently demonstrated lower absolute timing error than the CG. The effect sizes ranged from moderate-to-large (partial η^2^ = 0.197) to very large (partial η^2^ = 0.400), underscoring the robust and durable benefits of the deliberate practice intervention. Notably, the performance advantages and the corresponding effect sizes were more pronounced at the higher stimulus speed (10 mph), suggesting that the demanding nature of the training protocol was particularly effective in fine-tuning interceptive timing under increased spatiotemporal pressure.

### 3.7. Sensitivity Analyses

Given the violations of the homogeneity of variances observed in some of the models (indicated by Levene’s tests), supplementary analyses were conducted to ensure the validity of the primary findings. Bootstrap sensitivity analyses (utilizing 1000 resamples) and log-transformed ANCOVA models confirmed the robustness of the main group effects. As now explicitly reported in the main text and Table 2, the robust 95% Confidence Intervals demonstrated that the direction, magnitude, and statistical significance of the findings remained stable across all supplementary analyses (detailed in Supplementary Tables S1–S3), confirming that the observed differences were genuine effects of the intervention rather than statistical artifacts.

## 4. Discussion

### 4.1. Detailed Synthesis of Findings

This study investigated the longitudinal effects of an 8-week deliberate practice intervention on Coincidence Anticipation Timing (CAT) in 8–10-year-old female volleyball players. Results robustly supported our primary hypothesis, as the Experimental Group (EG) achieved significantly greater reductions in absolute timing error than the Control Group (CG) across both stimulus velocities (5 mph and 10 mph). Crucially, the EG maintained these gains over a two-month retention period, indicating a stable reorganization of the perceptual-motor system rather than transient performance fluctuations. Furthermore, the significantly larger effect size observed at the higher stimulus velocity (10 mph) provides critical insight into the hierarchical nature of motor control development in children.

### 4.2. Theoretical Underpinnings of Deliberate Practice in Youth Sports

The intervention’s success aligns with Ericsson et al.’s [37] framework of “deliberate practice.” Unlike “deliberate play,” which fosters general motor literacy, improving high-precision CAT in 8–10-year-olds demands a structured environment with high cognitive engagement and systematic error correction.

By progressively manipulating task constraints, from predictable trajectories to variable speeds and dual-task requirements, the intervention-maintained athletes in a state of “optimal challenge,” avoiding cognitive overload initially while increasing ecological pressure later. A pivotal factor in this process was the delivery of feedback. Immediate Knowledge of Results (KR) via millisecond-accurate displays enabled EG athletes to form a precise “reference of correctness.” This objective feedback loop is essential during late childhood when internal error-detection mechanisms are still maturing [30,32]. Consequently, the systematic reduction in timing error suggests the development of a refined “internal clock,” facilitating tighter synchronization between visual perception and motor initiation.

### 4.3. Neurophysiological Mechanisms: The Transition to Predictive Control

The velocity-dependent gains, evidenced by a larger effect size at 10 mph (partial η^2^ = 0.400) vs. 5 mph (partial η^2^ = 0.197), suggest the intervention specifically addressed limitations in reactive control.

Interceptive actions rely on two primary strategies: online tracking and predictive control [41]. At lower speeds (5 mph), the wider temporal window allows for continuous visual feedback and iterative movement corrections. Since this relies on slower feedback loops, the performance gap between trained and untrained individuals often narrows, explaining the CG’s respectable performance at 5 mph.

However, at 10 mph, Time-to-Contact (TTC) approaches the biological limits of human reaction time and neural processing delays, making online corrections impossible. Success here depends entirely on predictive control via “internal forward models” [42]. These models anticipate the target’s future state based on initial trajectory and speed. Our findings indicate that deliberate practice accelerated the maturation of these models. Repeated exposure to high-speed stimuli likely prompted a shift from a reactive “catch-up” mode to a proactive “predictive” mode [43]. We hypothesize this shift may be mediated by the functional refinement of cerebellar-cortical pathways, allowing young athletes to initiate motor commands earlier and effectively compensate for intrinsic sensorimotor delays [44].

### 4.4. Developmental Neuroplasticity and Motor Memory Consolidation

The durability of the CAT improvements, evidenced by the 2-month retention test, highlights the potential adaptability of the 8–10-year-old brain. This period is often described as the “golden age” of motor learning, characterized by a high degree of synaptic flexibility and an accelerated rate of motor consolidation [45].

While the present study did not collect direct neurophysiological data (e.g., EEG or fMRI), and therefore any assertions regarding specific neural pathways remain speculative, our behavioral retention data logically align with established models of predictive motor control maturation. Previous literature indicates that intensive, skill-based training in children can facilitate functional adaptations, such as optimized visual-motor pathway efficiency [46]. We cautiously hypothesize that our deliberate practice protocol may have engaged similar mechanisms. Unlike adults, who often require longer periods for “offline” consolidation, children demonstrate a unique ability to stabilize motor memories quickly and resist the interfering effects of other motor tasks [47,48]. The persistence of the EG’s superior timing accuracy after a two-month hiatus indicates that the “timing hardware” developed during the intervention was successfully encoded into long-term motor memory. Furthermore, the role of positive reinforcement and motivational feedback during the training may have been highly beneficial, as existing evidence highlights its critical role in supporting the consolidation of successful motor actions [49].

### 4.5. The Ecological Validity Gap: From the Lab to the Volleyball Court

Evaluating ecological validity and skill transferability is essential when interpreting laboratory-based outcomes. The Bassin Anticipation Timer, though a benchmark for measuring fundamental timing, utilizes a decoupled motor response (a button press) and a simplistic light stimulus, whereas actual volleyball environments present substantially more complex open-skill constraints [50]. On-court anticipation requires processing an opponent’s movements rather than generic visual stimuli; expert players extract advance kinematic cues—such as a server’s arm speed, a hitter’s approach angle, or a setter’s postural orientation—to guide their actions [2]. This refined perceptual-motor coupling distinguishes domain-specific expertise from general timing ability, explaining why light-based CAT improvements do not always directly translate into field-based reactive agility due to fundamental differences in informational variables [51].

Nevertheless, laboratory-based CAT can be conceptualized as the foundational “hardware” upon which sport-specific “software” is constructed; a child unable to accurately time a simple moving stimulus will likely struggle with the complex, multi-planar trajectory of a volleyball. Consequently, this 8-week intervention should be viewed as a foundational phase. To optimize skill transfer, future training protocols should transition toward representative learning designs [8]. This includes temporal occlusion training, where athletes predict trajectories from video clips cut off at the moment of contact—a method backed by meta-analytic evidence for enhancing on-court performance [52]. Additionally, integrating Virtual Reality (VR) could bridge this gap by combining laboratory-level control with realistic ball flights and whole-body interceptive responses [53].

### 4.6. Limitations and Future Directions

While the present findings are promising, several methodological constraints must be acknowledged. First, the sample was limited to female athletes within a narrow age range (8–10 years). Given that motor and cognitive development can vary significantly between sexes and during the onset of puberty, these results may not be directly generalizable to older cohorts or male athletes. Furthermore, the use of a button-press response on the Bassin Anticipation Timer, while necessary for millisecond precision in a controlled setting, lacks the full “perception-action coupling” of a whole-body athletic movement, such as a volleyball block or dig.

Second, the experimental group received a higher volume of specialized attention compared to the control group, which could theoretically introduce a “Hawthorne effect”—where performance improves simply because participants feel they are being observed or given special treatment. Additionally, while the duration of the training blocks was matched (24 min) for both groups, the density of active engagement likely differed. The generic technical drills of the CG inherently involve some wait time, whereas the EG’s deliberate practice drills demanded continuous, fast-paced cognitive and physical engagement. Therefore, the observed improvements might be partially attributed to higher practice volume and general arousal, rather than solely the CAT-specific task.

Relatedly, a potential theoretical risk of task-specific learning exists, given that a small fraction (<10%) of the experimental group’s training included visual light stimuli, while the primary assessment tool is also light-based. Rather than a complete ecological transfer to on-court volleyball skills, these findings reflect a partial transfer and a specific sensorimotor adaptation. We acknowledge that the shared stimulus modality—specifically the use of LED lights in both the reaction pods during training and the Bassin Timer during testing—undoubtedly facilitated the experimental group’s enhanced performance.

Future research should aim to replicate these findings with larger, more diverse samples and utilize portable technologies (e.g., wearable IMUs and eye-tracking) to measure timing during actual on-court actions, thereby enhancing the representative design of the experimental tasks. Finally, although some statistical assumptions regarding equal variances were violated, the use of robust estimation methods (i.e., bootstrapping with 1000 resamples) in our sensitivity analyses ensured that these minor deviations in data distribution did not compromise the validity of the reported intervention effects.

### 4.7. Practical Implications for Coaching and Talent Development

The practical implications for volleyball coaches are significant. Our study demonstrates that even a relatively short, 8-week structured intervention can meaningfully enhance a child’s timing capabilities. Coaches should consider integrating “timing-specific” blocks into their training sessions, moving beyond generic drills to tasks that explicitly demand predictive control.

For the 8–10 age group, the emphasis should be on high-frequency, high-feedback tasks. Instead of simply “playing the game,” young athletes benefit from drills where the outcome (success or failure in timing) is immediate and measurable. Furthermore, the velocity-dependent findings suggest that coaches should not be afraid to challenge young players with higher ball speeds, as these are the conditions that most effectively stimulate the development of advanced predictive strategies. By building this foundational timing “hardware” early, children will be better equipped to master the complex kinematic “software” of elite volleyball as they progress in their athletic careers.

## 5. Conclusions

In conclusion, the present study demonstrates that an 8-week deliberate practice intervention, characterized by structured goal-setting and high-density augmented feedback, significantly reduces coincidence anticipation timing errors in beginner female volleyball players aged 8–10 years. These performance gains were robustly maintained after a two-month retention period, particularly under the more demanding stimulus velocity condition (10 mph). While these findings highlight the practical value of embedding targeted cognitive-motor drills into regular club schedules, the scope of these conclusions is limited to the specific age cohort, sample size (*N* = 32), and sport modality examined. Within these boundaries, the intervention proved effective for enhancing and sustaining temporal prediction accuracy in youth athletes.

## Figures and Tables

**Table 1:** Descriptive statistics for CAT absolute error (ms) at 5 mph and 10 mph across time points for the Experimental Group (EG) and Control Group (CG).

Measure	EG *M* ± *SD*	CG *M* ± *SD*
Pre 5 mph	140 ± 76	137 ± 64
Post 5 mph	97 ± 44	127 ± 58
Retention 5 mph	89 ± 36	129 ± 60
Pre 10 mph	101 ± 63	122 ± 113
Post 10 mph	57 ± 37	129 ± 92
Retention 10 mph	58 ± 34	120 ± 86

Note: CAT = Coincidence Anticipation Timing; EG = Experimental Group (*n* = 16); CG = Control Group (*n* = 16); M = Mean; SD = Standard Deviation; ms = milliseconds; mph = miles per hour. All timing values represent absolute error, defined as the absolute difference between the stimulus arrival and the participant’s response. Lower values indicate higher timing accuracy. Pre = baseline assessment; Post = assessment immediately following the 8-week intervention; Retention = assessment conducted two months post-intervention.

**Table 2:** ANCOVA results for group differences in CAT absolute error at post-test and retention, controlling for baseline performance.

Outcome	Levene’s *p*	*F*(_1,29)_	*p*	Partial η^2^	Mean Difference (B)	Robust Bootstrapped 95% CI	EG Adjusted *M*	CG Adjusted *M*
Post 5 mph	0.028	7.122	0.012	0.197	−32 ms	[−57 to −9 ms]	96	128
Post 10 mph	0.002	19.336	<0.001	0.400	−58 ms	[−87 to −34 ms]	64	122
Retention 5 mph	0.109	13.179	0.001	0.312	−42 ms	[−66 to −19 ms]	88	130
Retention 10 mph	0.024	14.189	<0.001	0.329	−49 ms	[−80 to −24 ms]	65	114

Note: ANCOVA = Analysis of Covariance; CAT = Coincidence Anticipation Timing; EG = Experimental Group; CG = Control Group; Adjusted M = Estimated marginal means; partial η^2^ = partial eta squared (effect size); *F* = F-statistic. Degrees of freedom are shown in parentheses. Mean Difference (B) represents EG adjusted mean minus CG adjusted mean (negative values indicate lower error for the EG). Robust Bootstrapped 95% CI represents the 95% percentile confidence intervals derived from 1000 bootstrap resamples to confirm model stability under homoscedasticity violations.

## Data Availability

Data available on request due to restrictions (e.g., privacy, legal or ethical reasons).

## Supplementary Materials

The following supporting information can be downloaded at: https://www.mdpi.com/article/10.3390/children13060822/s1, Supplementary Table S1: Bootstrap ANCOVA; Supplementary Table S2: Linear Regression Sensitivity Analysis; Supplementary Table S3: Log Transformed ANCOVA.

## Abbreviations

The following abbreviations are used in this manuscript:

CAT	Coincidence Anticipation Timing
TTC	Time-to-Contact
KR	Knowledge of Results
KP	Knowledge of Performance
VEPs	Visual Evoked Potentials
LED	Light Emitting Diode
