# Supplementary material for: The Impact of an 8-Week Deliberate Practice Intervention on Coincidence Anticipation Timing and Long-Term Retention in Youth Female Volleyball Players
Source: Children (Basel). 2026 Jun 17;13(6):822. doi: 10.3390/children13060822 (PMC13298228; doi:10.3390/children13060822)
Supplement: Supplementary file 1 [file children-13-00822-s001.zip › children-4326207-supplementary.pdf]

Supplementary Materials

It must be separated to three separate files.

Supplementary Table S1.pdf; Supplementary Table S2.pdf; Supplementary Table S3.pdf

Supplementary Table S1. Bootstrap ANCOVA

Bootstrap ANCOVA Sensitivity Analysis for CAT Absolute Error at 5 mph (Post-test)

Overview

A bootstrap ANCOVA with 5,000 resamples was conducted to assess the robustness of the group effect on post-test CAT absolute error at 5 mph, controlling for baseline performance (pre-test CAT error). Percentile-based 95% confidence intervals were computed. The bootstrap analysis confirmed the stability of the parameter estimates obtained from the primary ANCOVA.

Bootstrap Specifications: Sampling method: Simple; Number of bootstrap samples: 5000; Confidence interval type: Percentile; *CI* level: 95%; Missing values: User-defined missing values excluded.

Table S1. Bootstrap Parameter Estimates for the ANCOVA Model (5 mph Post-test)

Parameter	B	Bias	Std. Error	Sig. (2- tailed)	95% *CI* Lower	95% *CI* Upper
Intercept	52	-0.01	17	0.007	18	84
Pre-test CAT	0.553	-6	133	<0.001	298	800
Group (EG vs CG)	-32	1	12	0.032	-57	-9

(Bootstrap results based on 5000 samples.)

Interpretation

The bootstrap ANCOVA confirmed that:
— The group effect remains statistically significant (p = .032).
— The confidence interval for the group effect does not cross zero (−57 to −9).
— The direction and magnitude of the effect are consistent with the primary ANCOVA.
— The covariate remains a strong and stable predictor of post-test performance.

These results demonstrate that the observed group difference at 5 mph is robust to sampling variability and not dependent on specific sample characteristics.

Supplementary Table S2. Linear Regression Sensitivity Analysis

Linear Regression Sensitivity Analysis for CAT Absolute Error at 5 mph (Post-test)

Overview

To evaluate the robustness of the ANCOVA findings for the 5 mph post-test condition, a linear regression model was conducted with post-test CAT absolute error as the dependent variable and group (EG vs. CG) and pre-test CAT error as predictors. This analysis served as a sensitivity check to examine whether the direction and magnitude of the group effect remained stable when estimated using an alternative modeling approach.

Model Summary

The overall model was statistically significant,
$\boldsymbol{F(2,29) = 22.726, p < .001}$,
explaining **61.0%** of the variance in post-test performance (R^2 = .610, Adjusted R^2 = .584).

Regression Coefficients

• Group (EG vs. CG): B = 32, *SE* = 12, β = 0.309, t = 2.669, *p* = .012
• Pre-test CAT error (5 mph): B = 0.553, *SE* = 88, β = 0.725, *t* = 6.252, *p* < .001

Model Diagnostics

• Durbin–Watson = 1.956 (no autocorrelation concerns)
• Collinearity: Tolerance = .999, VIF = 1.001 (no multicollinearity)
• Residuals: Standardized residuals −1.851 to 2.679 (acceptable)
• Influence: Maximum Cook's Distance = 1.033 (one case near threshold but no distortion of estimates)

Interpretation

The regression model confirmed that the group effect remained statistically significant and in the same direction as in the primary ANCOVA model, supporting the robustness of the primary results.

Supplementary Table S3. Log-Transformed ANCOVA

Log-Transformed ANCOVA for CAT Absolute Error at 5 mph (Post-test)

Overview

A log-transformed ANCOVA was conducted to evaluate whether the group effect at 5 mph remained stable after applying a variance-stabilizing transformation. The dependent variable was the natural logarithm of post-test CAT absolute error (lnPost), with group (Experimental vs. Control) as the fixed factor and the natural logarithm of pre-test CAT error (lnPre) as the covariate.

Model Summary

The log-transformed ANCOVA was statistically significant,
$F(2,29) = 40.411, p < .001$,
explaining **73.6%** of the variance in lnPost ($R^2 = .736$, Adjusted $R^2 = .718$).

Supplementary Table S3. Parameter Estimates for the Log-Transformed ANCOVA Model (5 mph Post-test)

**Parameter**	**B**	**Std. Error**	***t***	***p***	**95% CI Lower**	**95% CI Upper**	**Partial η^2**
Intercept	−0.537	0.203	−2.639	.013	−0.953	−0.121	.194
lnPre	0.766	0.092	8.289	<.001	0.577	0.955	.703
Group (EG vs CG)	−0.298	0.094	−3.158	.004	−0.491	−0.105	.256

Interpretation

The log-transformed ANCOVA confirmed that the group effect remained statistically significant and directionally consistent with the primary ANCOVA and bootstrap analyses. This log-transformed analysis was performed as a sensitivity check to ensure that the primary findings obtained on the original scale remain robust and stable after addressing potential variance heterogeneity. This indicates that the intervention effect at 5 mph is not dependent on variance heterogeneity and remains robust under transformation.
